# Politik zur Bewegungsförderung in Deutschland: Eine Analyse anhand eines Policy-Audit-Tools der Weltgesundheitsorganisation

**DOI:** 10.1007/s00103-021-03403-z

**Published:** 2021-08-27

**Authors:** Sven Messing, Sarah Forberger, Catherine Woods, Karim Abu-Omar, Peter Gelius

**Affiliations:** 1 Department für Sportwissenschaft und Sport, Friedrich-Alexander-Universität Erlangen-Nürnberg, Gebbertstraße 123 b, 91058 Erlangen, Deutschland; 2 Leibniz-Institut für Präventionsforschung und Epidemiologie – BIPS, Bremen, Deutschland; 3 University of Limerick, Limerick, Irland

**Keywords:** Bewegung, Public Health, Gesundheit, Politik, Audit, Physical activity, Public health, Health, Policy, Audit

## Abstract

**Hintergrund:**

Da Bewegungsmangel eine entscheidende Ursache für nichtübertragbare Erkrankungen ist, wurden sowohl von der Weltgesundheitsorganisation (WHO) als auch von Wissenschaftlerinnen und Wissenschaftlern weltweit verschiedene Tools zum Monitoring und Audit bewegungsfördernder Politik entwickelt. Allerdings wurden diese Tools bisher noch nicht genutzt, um systematisch und umfassend Daten zu bewegungsfördernder Politik in Deutschland zu erheben und zu analysieren.

**Ziel der Arbeit:**

Die Studie hat zum Ziel, die politischen Anstrengungen zur Bewegungsförderung in Deutschland in einem systematischen Überblick darzustellen.

**Material und Methoden:**

Die Erhebung wurde im Rahmen des Projekts „Policy Evaluation Network“ (www.jpi-pen.eu) unter Nutzung des Health-Enhancing Physical Activity (HEPA) Policy Audit Tools (PAT) der WHO durchgeführt. Datenquellen waren Erhebungen der WHO für das European Union Physical Activity Monitoring Framework, eigene Recherchearbeiten sowie eine Expertenbefragung.

**Ergebnisse:**

Die Ergebnisse zeigen das Spektrum der relevanten Akteure und bieten einen Überblick über aktuelle politische Maßnahmen sowie die Bereiche Surveillance, Evaluation und finanzielle Förderung. Darüber hinaus identifizieren sie wichtige Erfolge der aktuellen deutschen Politik sowie bestehende Herausforderungen.

**Diskussion:**

Im internationalen Vergleich fällt auf, dass andere Länder im Gegensatz zu Deutschland messbare nationale Ziele für die Förderung von Bewegung formuliert haben. Hervorzuheben ist, dass Deutschland zu der Minderheit der Staaten mit spezifischen Bewegungsempfehlungen für Menschen mit nichtübertragbaren Erkrankungen zählt. Eine besonders hohe Relevanz hat die Weiterentwicklung von Strukturen für Bewegungsförderung in Deutschland.

## Abstract (en)

**Background:**

Physical inactivity is a key determinant of noncommunicable diseases. Therefore, the World Health Organization (WHO) as well as researchers worldwide have developed different tools to monitor and audit policies to promote physical activity. However, these tools have so far not been used to systematically collect and analyse data on physical activity promoting policies in Germany.

**Aim:**

This study aims to provide a systematic overview of policies to promote physical activity in Germany.

**Methods:**

The study was conducted as part of the Policy Evaluation Network (www.jpi-pen.eu). Data from the European Union Physical Activity Monitoring Framework, desk research, and an expert survey were utilised and collected with the WHO’s health-enhancing physical activity (HEPA) policy audit tool (PAT).

**Results:**

The results highlight the wide range of relevant stakeholders and provide an overview of current policies as well as surveillance, evaluation, and funding. Significant accomplishments and persistent challenges are identified.

**Discussion:**

An international comparison shows that, in contrast to Germany, other countries have formulated measurable goals for physical activity promotion on a national level. However, Germany is among a minority of countries with specific physical activity recommendations for adults with noncommunicable diseases. Further structural development of physical activity promotion in Germany is necessary.

## Einleitung

Nichtübertragbare Erkrankungen, für deren Entstehung ein Mangel an Bewegung ein entscheidender Faktor sein kann [1], waren im Jahr 2019 weltweit für 41 Mio. vorzeitige Todesfälle verantwortlich [2]. Die Förderung von Bewegung ist daher nicht nur für die Weltgesundheitsorganisation (WHO) und die Europäische Union (EU) ein politisch relevantes Thema [3, 4]. Auch auf nationaler Ebene werden vermehrt politische Maßnahmen zur Bewegungsförderung verabschiedet, die die Wissenschaft unter anderem in vergleichenden Analysen untersucht [5–7].

Um Daten zu politischen Maßnahmen einzelner Länder im Bereich der Bewegungsförderung erheben zu können, wurden in den vergangenen Jahren verschiedene Monitoringinstrumente entwickelt. Dazu zählt unter anderem das Health-Enhancing Physical Activity (HEPA) Policy Audit Tool (PAT) der WHO [8], auf dessen Grundlage die EU ein regelmäßiges Monitoringsystem für Bewegungsförderung entwickelt hat [4]. Darüber hinaus existieren z. B. ein für den weltweiten Einsatz konzipiertes Monitoringframework für den Globalen Aktionsplan für Bewegung der WHO [9] sowie wissenschaftliche Instrumente zur umfassenden Analyse von Politik zur Bewegungsförderung [10]. Für Deutschland wurde die Politik zur Bewegungsförderung bisher jedoch noch nicht systematisch und umfassend im Sinne eines solchen Monitorings untersucht.

Um politische Entscheidungsträger zu informieren und die zukünftige Forschung zur Bewegungsförderungspolitik in Deutschland zu unterstützen, gibt dieser Artikel einen Überblick über aktuelle Strukturen, politische Dokumente und Maßnahmen der Bewegungsförderung auf nationaler Ebene. Hierdurch soll eine Art „Bestandsaufnahme“ gelingen, die auch Erfolge und Defizite identifiziert und eine Vergleichbarkeit der Politik in Deutschland mit anderen Nationen, in denen das HEPA PAT bereits angewendet wurde, erlaubt.

## Methoden

Ziel dieser Studie war eine systematische Erhebung von Daten zu bewegungsfördernder Politik in Deutschland. Diese erfolgte auf Grundlage des HEPA PATs der WHO [8]. Das HEPA PAT wurde zur Politikanalyse auf nationaler Ebene entwickelt, dient der standardisierten Datenerhebung und besteht aus einem Fragebogen mit 29 geschlossenen und offenen Fragen. Es bildet teilweise die Grundlage des EU Monitoring Frameworks für Bewegungsförderung [4], enthält darüber hinaus jedoch weitere Indikatoren, die eine detailliertere Erhebung von zusätzlichen Informationen ermöglichen. Die 29 Fragen des HEPA PATs sind inhaltlich 10 Themenbereichen zugeordnet, wie beispielsweise „Leadership und Partnerschaften“, „politische Dokumente“ oder „Empfehlungen und Ziele“. Eine detaillierte Übersicht über alle Themenbereiche findet sich in Tab. 1, ausgewählte Fragen werden in Tab. 2 dargestellt.

**Table 1:** 

Frage		Zentrale Datenquelle		
Nr.	Thema	Kategorie I (EU Physical Activity Monitoring Framework)	Kategorie II (eigene Recherche)	Kategorie III (Expertenbefragung)
*Hintergrundinformationen und landesspezifischer Kontext*				
1a	Struktur der Regierung	–	X	–
1b	Regierungssystem auf subnationaler Ebene	–	X	–
1c	Zentrale Ministerien der Regierung	–	X	–
1d	Andere wichtige nationale Organisationen	–	X	–
*Federführende Zuständigkeit und Partnerschaften*				
2	Leadership für Bewegungsförderung auf nationaler Ebene	X	–	–
3	Leadership für Bewegungsförderung auf subnationaler Ebene	–	X	X
4	Sektorübergreifende Zusammenarbeit auf nationaler Ebene	X	–	–
5	Sektorübergreifende Zusammenarbeit auf subnationaler Ebene	–	X	X
*Politische Dokumente*				
6	Zentrale politische Dokumente und Veranstaltungen der Vergangenheit	–	X	X
7	Zentrale politische Dokumente, Gesetzgebung, Strategien und Aktionspläne der Gegenwart	X	X	X
8	Verwendung eines konsultativen Prozesses	X	X	–
9	Evidenz für Querverweise und Angleichung innerhalb von und zwischen verschiedenen Politiken	–	X	X
10	Evidenzbasis der zentralen politischen Dokumente	–	X	–
11	Nützlichkeit von internationalen Dokumenten	–	–	X
12	Nationale Dokumente oder Richtlinien zur Unterstützung der Implementierung von bewegungsfördernden Aktivitäten auf subnationaler Ebene	–	X	X
*Politikbereiche, -inhalte und -implementierung*				
13	Lebenswelten von bewegungsfördernden Aktivitäten	X	X	–
14	Zielgruppen für bewegungsfördernde Aktivitäten	X	X	–
15	Nationale (massenmediale) Kommunikationsstrategie	X	–	–
16	Beispiele für groß angelegte Programme	–	X	X
*Empfehlungen und Ziele*				
17a	Nationale Empfehlungen für Bewegung und Gesundheit	X	X	–
17b	Nationale Empfehlungen zur Reduktion von sitzenden Verhaltensweisen	X	X	–
18	Nationale Ziele für die Prävalenz der Bevölkerung hinsichtlich Bewegung	X	X	–
19	Andere Ziele mit direktem oder indirektem Bezug zu Bewegung	X	X	–
*Surveillance*				
20	Surveillance- oder Monitoringsystem für Gesundheit	X	X	–
21a	Einfluss von Daten zur Prävalenz von Bewegung oder sitzendem Verhalten auf Politikentwicklung	–	–	X
21b	Einfluss von Surveillance-Daten auf die Bewegungsförderung auf nationaler Ebene	–	–	X
*Evaluation*				
22a	Evaluation von nationaler Politik oder Aktionsplänen	X	–	–
22b	Evaluation auf subnationaler Ebene	–	X	X
23	Ökonomische Evaluation von Interventionen oder von körperlicher Inaktivität	–	X	–
*Finanzielle Förderung und Commitment*				
24a	Finanzielle Förderung auf nationaler Ebene	X	–	–
24b	Finanzielle Förderung auf subnationaler Ebene	–	X	X
25	Politisches Commitment	–	–	X
*Kapazitätsentwicklung durch ein nationales Netzwerk*				
26	Netzwerk oder System zur Vernetzung und/oder Unterstützung von Fachleuten	–	X	–
*Erfahrung durch Politikimplementierung, Erfolge und verbleibende Herausforderungen*				
27a	Größte Erfolge in der nationalen Bewegungsförderung	–	–	X
27b	Größte Herausforderungen in der nationalen Bewegungsförderung	–	–	X
28	Vorschläge für andere Länder	–	–	X
29	Weitere Details oder Kommentare	–	X	–

**Table 2:** 

Nr.	Thema	Frage (übersetzt aus dem Englischen)
1c	Zentrale Ministerien der Regierung	Bitte erstellen Sie eine Liste der zentralen Ministerien der Regierung auf nationaler Ebene, die eine Rolle in der Bewegungsförderung spielen (z. B. Gesundheit, Sport, Bildung, Transport, Umwelt und Stadtplanung)
2	Leadership für Bewegungsförderung auf nationaler Ebene	Bitte nennen Sie die Behörde(n), die in Ihrem Land auf nationaler Ebene federführend für Bewegungsförderung zuständig ist (bzw. sind)
4	Sektorübergreifende Zusammenarbeit auf nationaler Ebene	Gibt es in Ihrem Land Mechanismen oder Behörden, die eine sektorübergreifende Zusammenarbeit in der Politik zur Bewegungsförderung auf nationaler Ebene sicherstellen?
		Falls ja, beschreiben Sie diese kurz. Bitte stellen Sie Informationen zur Verfügung, wer involviert ist, wer diese Anstrengungen leitet und wie diese Zusammenarbeit in der Praxis funktioniert. Bitte erwähnen Sie auch (soweit möglich) positive oder negative Erfahrungen. Dies kann auch Beispiele der Zusammenarbeit mit privaten oder ehrenamtlichen Sektoren einschließen
7	Zentrale politische Dokumente, Gesetzgebung, Strategien und Aktionspläne der Gegenwart	Bitte beschreiben Sie die aktuellen zentralen politischen Dokumente, Gesetzgebung, Strategien oder Aktionspläne in Ihrem Land, in denen die Absicht der Regierung (und ggf. von Nichtregierungsorganisationen) zur Bewegungsförderung dargestellt ist, im Detail (Titel, Zeitraum, herausgebende Organisation)
		Bitte listen Sie die Dokumente nach Sektoren auf und geben Sie, sofern verfügbar, einen Weblink an (einschließlich einer Angabe, ob eine englische Version oder Zusammenfassung verfügbar ist). Bitte beschreiben Sie den Inhalt jeder Politik kurz (ca. 100–250 Wörter)
		Bitte markieren Sie in der rechten Spalte, welche Dokumente am wichtigsten für Bewegungsförderung in Ihrem Land sind, und erklären Sie kurz, warum diese Dokumente als wichtig erachtet werden
28	Vorschläge für andere Länder	Auf Grundlage Ihrer Erfahrung, bitte identifizieren Sie bis zu 3 Vorschläge, die Sie einem anderen Land machen würden, das eine nationale Politik zur Bewegungsförderung aufbaut

Diese Studie wurde als Teil des von der Joint Programming Initiative „A Healthy Diet for a Healthy Life“ geförderten Projekts „Policy Evaluation Network“ (PEN) durchgeführt, in dem 28 Forschungsinstitutionen aus 7 europäischen Staaten und Neuseeland miteinander kooperieren (www.jpi-pen.eu). In Deutschland fand die Datenerhebung von März bis Oktober 2019 statt und wurde von Wissenschaftlerinnen und Wissenschaftlern der Friedrich-Alexander-Universität Erlangen-Nürnberg (FAU) sowie des Leibniz-Instituts für Präventionsforschung und Epidemiologie Bremen (BIPS) durchgeführt. Analog dazu wurden Daten in Irland, den Niederlanden und Polen erhoben. Während die Ergebnisse dieses 4‑Länder-Vergleichs an anderer Stelle publiziert wurden [7], bereitet dieser Artikel die deutschen Ergebnisse in einer größeren Detailtiefe auf.

Für die Studie wurde das HEPA PAT in Abstimmung mit den internationalen Partnern des PEN-Forschungsnetzwerks in 3 Kategorien unterteilt (Tab. 1). Die beteiligten Wissenschaftlerinnen und Wissenschaftler vervollständigten zunächst jene Teile des Fragebogens, die anhand der Daten des EU Physical Activity Monitoring Framework beantwortet werden konnten (Kategorie I). Diese Informationen wurden 2018 von der EU und dem WHO-Regionalbüro für Europa durch eine Befragung der offiziellen Ansprechpersonen (Physical Activity Focal Points) aller EU-Regierungen erhoben und dem PEN-Projekt für die Studie zur Verfügung gestellt. Anschließend wurde eine gezielte Internetrecherche durchgeführt, unterstützt durch die Kontaktaufnahme mit einzelnen Organisationen, um spezifische Informationen zu verifizieren oder zu ergänzen (Kategorie II). Im Anschluss beteiligten sich 13 Mitglieder der Arbeitsgruppe „Bewegungsförderung im Alltag“ des Bundesministeriums für Gesundheit an einer Onlineumfrage. Hierdurch konnten jene Teile des HEPA PATs komplettiert werden, die einer Experteneinschätzung bedürfen (Kategorie III). Abschließend führte die Forschungsgruppe alle Informationen zusammen und füllte das HEPA PAT formal für Deutschland aus. Wir stellen nachfolgend die Ergebnisse zur Politik auf nationaler Ebene dar, strukturiert anhand der Themenbereiche des HEPA PAT (Tab. 1).

## Ergebnisse

### Hintergrundinformationen und landesspezifischer Kontext sowie federführende Zuständigkeit und Partnerschaften

Das HEPA PAT sammelt zunächst wichtige Informationen über die Strukturen der Bewegungsförderung in einem Land. Für Deutschland ist das Bundesministerium für Gesundheit auf nationaler Ebene federführend für Bewegungsförderung zuständig und wird dabei vom Robert Koch-Institut und der Bundeszentrale für gesundheitliche Aufklärung unterstützt. Eine zentrale Rolle für die Koordination in diesem Bereich auf Bundesebene übernimmt die Arbeitsgruppe „Bewegungsförderung im Alltag“ des Bundesministeriums für Gesundheit. Diese Arbeitsgruppe umfasst Mitarbeiterinnen und Mitarbeiter von Ministerien, wissenschaftlichen Fachgesellschaften und Verbänden sowie weitere Expertinnen und Experten aus den Bereichen Gesundheitsförderung, Prävention und Sport; eine vollständige Liste der Arbeitsgruppenmitglieder ist im Impressum der Nationalen Empfehlungen für Bewegung und Bewegungsförderung zu finden [11]. Weitere für Bewegungsförderung relevante staatliche Akteure sind das Bundesministerium des Innern, für Bau und Heimat, das Bundesministerium für Verkehr und digitale Infrastruktur, das Bundesministerium für Familie, Senioren, Frauen und Jugend, das Bundesministerium für Umwelt, Naturschutz und nukleare Sicherheit sowie das Bundesministerium für Bildung und Forschung. Auch die Bundesländer haben relevante Kompetenzen, insbesondere in den Kultus‑, Sport- und Gesundheitsministerien, auch hinsichtlich der Ausführung von Bundesgesetzen. Daher sind auf Bundesebene die entsprechenden Landesministerien sowie ihre jeweiligen Gremien, die Kultus‑, Sport- und Gesundheitsministerkonferenz, von Relevanz. Darüber hinaus spielt auch die kommunale Ebene eine wichtige Rolle in der Bewegungsförderung, z. B. bei der Förderung von Sportvereinen und des Schulsports, bei der Planung von Bewegungs- und Erholungsräumen sowie beim Fuß- und Radwegebau. Aus diesem Grund sind die kommunalen Spitzenverbände wie der Deutsche Städtetag, der Deutsche Landkreistag und der Deutsche Städte- und Gemeindebund ebenfalls von Bedeutung. Die zentralen staatlichen Akteure für Bewegungsförderung sind in Tab. 3 zusammenfassend dargestellt.

**Table 3:** 

Politische Ebene	Politischer Sektor	Zentrale staatliche Akteure	Bezug zu Bewegungsförderung (Beispiele)
Bund	Gesundheit	Bundesministerium für Gesundheit	Federführende Zuständigkeit des Bundesministeriums für Gesundheit, koordinierende Rolle der Arbeitsgruppe „Bewegungsförderung im Alltag“
		Robert Koch-Institut	
		Bundeszentrale für gesundheitliche Aufklärung	
		Arbeitsgruppe „Bewegungsförderung im Alltag“ des Bundesministeriums für Gesundheit	
	Sport und Stadtplanung	Bundesministerium des Innern, für Bau und Heimat	Leistungssport, Stadtplanung
	Verkehr	Bundesministerium für Verkehr und digitale Infrastruktur	Aktiver Transport
	Familie	Bundesministerium für Familie, Senioren, Frauen und Jugend	Bewegungsförderung im Kindergarten
	Umwelt	Bundesministerium für Umwelt, Naturschutz und nukleare Sicherheit	Naturräume
	Bildung	Bundesministerium für Bildung und Forschung	Forschungsförderung
Bundesländer	Gesundheit	Gesundheitsministerkonferenz und -ministerien	Federführende Zuständigkeit auf Landesebene
	Sport	Sportministerkonferenz und -ministerien	Breitensport
	Bildung	Kultusministerkonferenz und -ministerien	Bewegungsförderung in Schulen
Kommunen	Verschiedene Sektoren	Deutscher Städtetag	Förderung von Sportvereinen und des Schulsports, Planung von Bewegungs- und Erholungsräumen, Fuß- und Radwegebau
		Deutscher Landkreistag	
		Deutscher Städte- und Gemeindebund	

### Politische Dokumente

Wichtige politische Dokumente in der Bewegungsförderung in Deutschland wurden im Gesundheits‑, Verkehrs- und Stadtplanungssektor identifiziert (Tab. 4). Als zentral gelten der Nationale Aktionsplan IN FORM zur Prävention von Fehlernährung, Bewegungsmangel, Übergewicht und damit zusammenhängenden Krankheiten [12], das Präventionsgesetz [13], die Nationalen Empfehlungen für Bewegung und Bewegungsförderung [11] sowie der Nationale Radverkehrsplan 2020 [14]. Bei der Politikentwicklung spielten konsultative Prozesse in der Regel eine wichtige Rolle. Beispielsweise war die Entwicklung des Nationalen Aktionsplans IN FORM zwar eine politische Entscheidung, allerdings wurde bereits zu einem frühen Zeitpunkt eine Nationale Steuerungsgruppe zur Beratung der zuständigen Bundesministerien eingerichtet, über die neben den Ländern und Kommunen auch wesentliche zivilgesellschaftliche Akteure eingebunden wurden. Es wurden verschiedene Querverweise zwischen den politischen Dokumenten identifiziert. Auffällig ist, dass sich die wichtigsten Dokumente alle auf IN FORM beziehen – auch in der Gesetzesbegründung zum Präventionsgesetz findet sich ein entsprechender Verweis [15]. Ein Beispiel für die Evidenzbasierung der Politik sind die Nationalen Empfehlungen für Bewegung und Bewegungsförderung, die auf Grundlage wissenschaftlicher Studien entwickelt wurden [11, 16].

**Table 4:** 

Politischer Sektor	Dokument	Jahr
Gesundheit	Nationaler Aktionsplan IN FORM^a^ [12]	2008
	Präventionsgesetz^a^ [13]	2015
	Nationale Empfehlungen für Bewegung und Bewegungsförderung^a^ [11]	2016
	Bundesrahmenempfehlungen der Nationalen Präventionskonferenz [34]	2016
	Leitfaden Prävention des GKV-Spitzenverbands [35]	2018 (ursprünglich 2000)
Verkehr	Nationaler Radverkehrsplan 2020^a^ [14]	2012
Stadtplanung	Forschungsprogramm „Experimenteller Wohnungs- und Städtebau“ (ExWoSt; [36])	1987
	Programm „Soziale Stadt“ [37]	1999

^a^ Wichtigste Dokumente

### Politikbereiche, -inhalte und -implementierung

Die zentralen politischen Dokumente beziehen sich auf nahezu alle Lebenswelten und Bevölkerungsgruppen, die im HEPA PAT abgefragt werden. Konkret umfasst dies die Bereiche Schule, Arbeitsplatz und die medizinische Versorgung sowie Kinder und Jugendliche, Frauen und sozial benachteiligte Menschen. Lediglich der Tourismussektor scheint kein Bereich der nationalen Politik zur Bewegungsförderung zu sein.

Obwohl es in Deutschland keine nationale massenmediale Kommunikationsstrategie zur Bewegungsförderung gibt, existieren vielfältige Maßnahmen zur Bewusstseinssteigerung. Dazu zählen beispielsweise verschiedene Aktivitäten im Rahmen von IN FORM, Materialien zur Dissemination der Nationalen Empfehlungen für Bewegung und Bewegungsförderung [17] sowie Aufklärungsmaßnahmen der Bundeszentrale für gesundheitliche Aufklärung, z. B. zur Prävention von Kinderübergewicht [18].

### Empfehlungen und Ziele

In Deutschland gibt es Nationale Empfehlungen für Bewegung und Bewegungsförderung für 4 der 5 im HEPA PAT genannten Zielgruppen: Kleinkinder, Schulkinder und Jugendliche, Erwachsene und ältere Erwachsene [11]. Darüber hinaus hat Deutschland spezifische Empfehlungen für Erwachsene mit einer nichtübertragbaren Erkrankung formuliert und gehört somit zu den weniger als 50 % der Staaten mit Bewegungsempfehlungen für diese Zielgruppe [6, 19]. Von den im HEPA PAT explizit abgefragten Zielgruppen gibt es in Deutschland zum jetzigen Zeitpunkt lediglich für Menschen mit Behinderung keine spezifischen Empfehlungen.^1^

Im Nationalen Aktionsplan IN FORM ist das Ziel festgelegt, das Bewegungsverhalten der Menschen in Deutschland nachhaltig zu verbessern und bis 2020 sichtbare Ergebnisse zu erreichen [12]. Auch im Präventionsgesetz ist die Förderung von Bewegung als Gesundheitsziel verankert [13]. Konkretere Ziele zur Steigerung der Bewegungsprävalenz der Bevölkerung wurden in den zentralen nationalen politischen Dokumenten jedoch nicht formuliert. Durch die Verabschiedung des „Global Action Plan for the Prevention and Control of Noncummunicable Diseases 2013–2020“ der WHO hat sich Deutschland allerdings dazu bekannt, die Prävalenz von unzureichender Bewegung bis 2025 um 10 % zu reduzieren [3].

Als weiteres politisches Ziel mit Bezug zu Bewegung formuliert der Nationale Radverkehrsplan, den Anteil des Radverkehrs bis 2020 signifikant zu steigern [14].^2^ Darüber hinaus verweist das Dokument auf Ziele aus anderen politischen Sektoren, wie beispielsweise die im Energiekonzept der Bundesregierung verankerte Reduzierung des Energieverbrauchs im Verkehrssektor um 10 % bis 2020 (im Vergleich zu 2005; [20]).

### Surveillance

Im Rahmen des bundesweiten Gesundheitsmonitorings in Deutschland [21] führt das Robert Koch-Institut regelmäßig Gesundheitsbefragungen wie die Studie zur Gesundheit von Kindern und Jugendlichen in Deutschland (KiGGS; [22]), die Studie zur Gesundheit Erwachsener in Deutschland (DEGS; [23]) und die Studie „Gesundheit in Deutschland aktuell“ (GEDA; [24]) durch. Außerdem betonten die befragten Expertinnen und Experten die Relevanz der Eurobarometer-Daten (z. B. [25]) und der Nichtbeweger-Studie der BARMER [26].

Die überwiegende Mehrheit der von uns befragten Expertinnen und Experten (85 %) gab an, dass solche Daten zur Bewegungsprävalenz verwendet wurden, um nationale Ziele zu formulieren oder um den Fortschritt zur Erreichung der nationalen Ziele zu bewerten; beispielsweise hinsichtlich der zukünftigen Strategie des Nationalen Aktionsplans IN FORM sowie als Basisinformation für politische Aktivitäten in Zusammenhang mit den Nationalen Empfehlungen für Bewegung und Bewegungsförderung. Fast alle Befragten (92 %) gaben außerdem an, dass Surveillance-Daten auf andere Weise dabei geholfen haben, die Bewegungsförderung in Deutschland auf nationaler Ebene zu verbessern. Dies wurde unter anderem mit der gestiegenen Aufmerksamkeit für das Thema in Politik, Gesellschaft und Medien begründet.

### Evaluation

2 der 4 zentralen politischen Maßnahmen zur Bewegungsförderung wurden bereits evaluiert: Der Abschlussbericht zur Evaluation des Nationalen Aktionsplans IN FORM wurde im Oktober 2019 veröffentlicht [27]. Der Nationale Radverkehrsplan wurde ebenfalls im Jahr 2019 evaluiert, die Ergebnisse sind bislang allerdings noch nicht veröffentlicht [28]. Eine Evaluation der Nationalen Empfehlungen für Bewegung und Bewegungsförderung ist zum jetzigen Zeitpunkt nicht geplant.

Als ökonomische Evaluation liegt aktuell eine Studie vor, die die Kosteneffektivität von bewegungsfördernden Interventionen für Deutschland analysiert. Diese wurde von der Berliner Senatsverwaltung für Inneres und Sport im Jahr 2016 unter dem Titel „Bewegung fördern zahlt sich aus. Zum ökonomischen Nutzen von Bewegungsförderung“ [29] veröffentlicht. Der Transfer von Forschungsergebnissen aus anderen Ländern auf Deutschland kam unter anderem zu dem Ergebnis, dass politische Maßnahmen zur Förderung des Radverkehrs einen volkswirtschaftlichen Nutzen von 5,6 Mrd. € pro Jahr generieren könnten.

### Finanzielle Förderung und Commitment

Auf nationaler Ebene gibt es in Deutschland verschiedene finanzielle Fördermöglichkeiten für politische Maßnahmen und Aktionspläne zur Bewegungsförderung, unter anderem im Gesundheits‑, Sport‑, Bildungs‑, Verkehrs‑, Stadtplanungs- und Umweltsektor. Ein exakter Betrag ließ sich aufgrund der Vielfalt der Mechanismen nicht ermitteln. Beispiele sind das Präventionsgesetz mit über 500 Mio. €/Jahr (nicht ausschließlich für Bewegungsförderung) und der Förderschwerpunkt Bewegung im Nationalen Aktionsplan IN FORM mit 4,6 Mio. € für den Zeitraum 2019–2022.

Ein Großteil der befragten Expertinnen und Experten (77 %) war überzeugt, dass es in Deutschland politisches Commitment zur Förderung von Bewegung gibt. Dies begründeten sie unter anderem mit dem Nationalen Aktionsplan IN FORM, Aktivitäten in Zusammenhang mit den Nationalen Empfehlungen für Bewegung und Bewegungsförderung, diversen Fördermöglichkeiten sowie der Diskussion um ein nationales Präventionsziel für Bewegung. Allerdings sahen die Befragten Potenzial für noch mehr politisches Commitment und betonten, dass die Förderung von Bewegung nicht in allen relevanten Sektoren eine substanzielle Rolle spiele. Darüber hinaus bliebe die nachhaltige Implementierung von Aktivitäten zur Bewegungsförderung häufig schwierig, da viele Maßnahmen auf Projektbasis lediglich für einen begrenzten Zeitraum gefördert würden.

### Kapazitätsentwicklung durch ein nationales Netzwerk

Wir identifizierten in der vorliegenden Studie verschiedene Organisationen und Netzwerke, die Fachleute für Bewegungsförderung vernetzen und unterstützen. Dazu zählen insbesondere der Deutsche Olympische Sportbund (DOSB, www.dosb.de), einschließlich seiner Spitzenverbände und Landessportbünde, die Deutsche Gesellschaft für Sportmedizin und Prävention (DGSP, www.dgsp.de), der Deutsche Verband für Gesundheitssport und Sporttherapie (DVGS, www.dvgs.de), die Deutsche Vereinigung für Sportwissenschaft (dvs, www.sportwissenschaft.de), die Deutsche Gesellschaft für Sozialmedizin und Prävention (DGSMP, www.dgsmp.de) und die Deutsche Gesellschaft für Prävention und Gesundheitsförderung (DGPG, www.dg-pg.de).

### Erfahrungen durch Politikimplementierung, Erfolge und Herausforderungen

Von den befragten Expertinnen und Experten bewerteten 69 % die Veröffentlichung wissenschaftlich fundierter Nationaler Empfehlungen für Bewegung und Bewegungsförderung als einen der größten Erfolge in diesem Politikfeld in den vergangenen Jahren. Weitere häufig genannte Erfolge waren die Ermöglichung von spezifischen Forschungsaktivitäten für Bewegungsförderung durch die Bundesministerien (46 %), die Verabschiedung des Präventionsgesetzes (38 %) sowie der Nationale Aktionsplan IN FORM (23 %).

Als größte Herausforderung nannten die Mitglieder der Arbeitsgruppe „Bewegungsförderung im Alltag“ die Strukturen der Bewegungsförderung in Deutschland. Dies betrifft einerseits die staatliche Struktur wie die zu Beginn des Ergebnisteils dargestellte Kompetenzverteilung zwischen den verschiedenen Ebenen im föderalen System und zwischen den verschiedenen Ministerien sowie die intersektorale Zusammenarbeit. Andererseits wiesen sie auch auf fehlende Strukturen im nichtstaatlichen Bereich hin, die z. B. zur Zusammenführung von Aktivitäten und dem Interessensausgleich zwischen zivilgesellschaftlichen Akteuren beitragen könnten. Als weitere große Herausforderung erscheint zudem die geringe öffentliche Aufmerksamkeit für das Thema Bewegungsförderung im Vergleich zu anderen Handlungsfeldern in der Prävention und Gesundheitsförderung.

Die Expertinnen und Experten formulierten auf Basis ihrer Erfahrungen in Deutschland außerdem eine Reihe konkreter Vorschläge für andere Länder, die eine nationale Politik zur Bewegungsförderung etablieren möchten: Erstens sollten diese Länder relevante Stakeholder von Beginn an in die Politikentwicklung einbeziehen, beispielsweise durch die Bildung eines Steuerungsgremiums auf nationaler Ebene. Zweitens rieten sie zur Verabschiedung eines settingübergreifenden und evidenzbasierten Nationalen Aktionsplans. Und drittens wurde angeregt, nationale Bewegungsempfehlungen als „Aktivierungsansatz“ zur Stimulierung konkreter Maßnahmen und Interventionen zu nutzen.

## Diskussion

### Deutschland im internationalen Vergleich

Im internationalen Vergleich weist die Politik zur Bewegungsförderung in Deutschland verschiedene Besonderheiten auf. Eine kürzlich erschienene vergleichende Analyse zeigt, dass die meisten EU-Staaten zwar Bewegungsempfehlungen für Kinder und Jugendliche sowie für Erwachsene verabschiedet hatten und in 18 Ländern auch Empfehlungen für ältere Erwachsene existieren [6]. Spezifische Empfehlungen für Menschen mit nichtübertragbaren Erkrankungen wie in Deutschland gibt es dagegen nur in 8 weiteren EU-Mitgliedstaaten [6]. In Zukunft könnte Deutschland Bewegungsempfehlungen für Menschen mit Behinderung entwickeln, die immerhin in 9 europäischen Ländern bereits existieren [6]. Daneben fällt auf, dass andere Länder messbare nationale Ziele für Bewegungsförderung formuliert haben. Beispielsweise streben die Niederlande an, dass 75 % der Bevölkerung bis zum Jahr 2040 die Bewegungsempfehlungen erfüllen sollen [7]. Deutschland hat dagegen bislang kein vergleichbares messbares Ziel auf nationaler Ebene formuliert, was sich möglicherweise auch durch die vielfältigen Kompetenzen der Bundesländer in den entsprechenden Politikfeldern erklären lässt. In einer vergleichenden Analyse von 7 europäischen Ländern (ohne Deutschland) wurde allerdings schon vor einigen Jahren festgestellt, dass der Erfolg oder Misserfolg von nationaler Politik zur Bewegungsförderung ohne messbare Ziele oder konkrete Zeitrahmen für die Zielerreichung nicht evaluiert werden kann [5].

Ähnlichkeiten zu anderen europäischen Ländern gibt es hinsichtlich massenmedialer Kampagnen. Diese sind auch in anderen Ländern häufig mit spezifischen Initiativen verknüpft, anstatt auf übergreifende Kampagnen zur Bewegungsförderung zu setzen [5]. Somit ist es im internationalen Vergleich nicht ungewöhnlich, dass es in Deutschland keine übergreifende massenmediale Kommunikationsstrategie auf nationaler Ebene gibt. Auch die Verankerung der Bewegungsförderung im Gesundheitsministerium ist international durchaus üblich. Eine vergleichende Analyse von Bull et al. aus dem Jahr 2014 identifizierte Ministerien für Gesundheit als diejenigen, die am häufigsten die Führung und Koordination in Bezug auf Bewegungsförderung übernehmen [5]. Eine neuere Veröffentlichung zeigt hingegen, dass in manchen Ländern das Sportministerium auf nationaler Ebene zuständig ist oder die Kompetenzen zwischen dem Gesundheits‑, Sport- und Verkehrssektor geteilt sind [7].

### Politische Strukturen der Bewegungsförderung in Deutschland

Ein wichtiges Ergebnis dieser Forschungsarbeit ist, dass aus Sicht der befragten Expertinnen und Experten die Strukturen der Bewegungsförderung in Deutschland eine zentrale Herausforderung in diesem Politikfeld darstellen. Sowohl im staatlichen als auch im nichtstaatlichen Bereich könnten strukturelle Verbesserungen zu Synergieeffekten führen, beispielsweise im Bereich der intersektoralen Zusammenarbeit oder der Koordinierung der Aktivitäten zivilgesellschaftlicher Akteure. Aus einer übergeordneten Perspektive könnten verbesserte Strukturen unter anderem dazu beitragen, einen Health-in-All-Policies-Ansatz [30] in Bezug auf Bewegungsförderung systematisch umzusetzen. Zukünftige Forschungsarbeiten könnten ebenfalls einen Fokus auf die Strukturen der Bewegungsförderung in Deutschland legen und Optionen für deren Weiterentwicklung diskutieren.

### Relevanz von Ländern und Kommunen

Eine international vergleichende Analyse kommt zu dem Ergebnis, dass Gesetze zur Bewegungsförderung am häufigsten im Bildungssektor erlassen wurden, insbesondere mit Blick auf verpflichtenden Sportunterricht in Schulen [5]. In diesem Zusammenhang erscheint es zunächst überraschend, dass für Deutschland keine politischen Maßnahmen im Bildungssektor identifiziert werden konnten. Dies kann jedoch durch das föderale System und die Kompetenzen der Bundesländer in der Bildungspolitik leicht erklärt werden. Somit wäre für Deutschland eine weitere Analyse bewegungsfördernder Politik auf Länderebene bzw. im Rahmen der Kultus‑, Gesundheits- und Sportministerkonferenz notwendig, um ein vollständigeres Bild zur bewegungsfördernden Politik zu erreichen. Eine solche subnationale Untersuchung mithilfe eines modifizierten HEPA PATs wurde beispielsweise bereits in Japan durchgeführt [31].

Darüber hinaus ist auch die Rolle der Kommunen in der Bewegungsförderungspolitik von Bedeutung. Diese leisten unter anderem im Rahmen der kommunalen Sportentwicklungsplanung einen wichtigen Beitrag zur Umsetzung von nationaler und sogar internationaler Politik [32]. Eine flächendeckende Erhebung zu den Aktivitäten der deutschen Kommunen in der Bewegungsförderung wäre daher wünschenswert. Ein möglicherweise geeignetes Instrument ist in Frankreich entwickelt und exemplarisch eingesetzt worden [33].

### Limitationen

Durch den Fokus des HEPA PATs auf die nationale Ebene konnten vor allem jene Politikbereiche zur Bewegungsförderung in Deutschland, die auch von den Bundesländern und Kommunen mitgestaltet werden, nicht umfassend abgebildet werden. Darüber hinaus waren die zeitlichen und personellen Ressourcen für die Forschung begrenzt, sodass sich beispielsweise exaktere Summen aller verfügbaren finanziellen Fördermöglichkeiten für Bewegung nicht ermitteln ließen. Eine weitere Limitation ist, dass das HEPA PAT aufgrund seiner allgemeinen Perspektive einzelne relevante Aspekte – wie beispielsweise die Bewegungsförderung sozial benachteiligter Zielgruppen, die besonders häufig von Bewegungsmangel betroffen sind – nur punktuell erfasst. Dennoch bietet das HEPA PAT einen guten Überblick zur Bewegungsförderungspolitik in Deutschland, der aufgrund der Standardisierung des Tools international vergleichbar ist.

### Schlussfolgerungen

In Deutschland existieren auf nationaler Ebene bereits zahlreiche politische Maßnahmen zur Bewegungsförderung. Im internationalen Vergleich wird allerdings deutlich, dass es neben Erfolgen in verschiedenen Bereichen weiteres Potenzial für eine bewegungsfördernde Politik gibt: Dies betrifft z. B. spezifische Bewegungsempfehlungen für Menschen mit Behinderung oder die Formulierung messbarer nationaler Ziele für die Bewegungsförderung. Von besonders hoher Relevanz ist die zukünftige Weiterentwicklung der Strukturen für Bewegungsförderung in Deutschland. Im Bereich der Forschung erscheinen insbesondere systematische Erhebungen der Aktivitäten auf Länderebene und in den Kommunen sinnvoll, um ein vollständiges Bild der Politik zur Bewegungsförderung in Deutschland zeichnen zu können.

## Footnotes

1 Die Nationalen Empfehlungen für Bewegung und Bewegungsförderung beinhalten solche Empfehlungen nicht, betonen aber deren Notwendigkeit (ebenso wie für sozial benachteiligte Menschen und schwangere Frauen sowie genderspezifische Empfehlungen).

2 Nach Abschluss der Datenerhebung für das HEPA PAT wurde im April 2021 der Nationale Radverkehrsplan 3.0 von der Bundesregierung beschlossen. Dieser beinhaltet deutlich konkretere Ziele wie eine Steigerung der Anzahl der mit dem Fahrrad zurückgelegten Wege pro Person und Jahr von 120 (2017) auf 180 (2030) und eine Steigerung der durchschnittlichen Wegelänge von 3,7 km (2017) auf 6 km (2030; [38]).
